# Effect of Preoperative Sarcopenic Obesity on Outcomes in Patients with Gastric Cancer After Surgery

**DOI:** 10.3390/cancers18020191

**Published:** 2026-01-07

**Authors:** Itaru Hashimoto, Keisuke Komori, Norihiro Akimoto, Yuta Nakayama, Shinsuke Nagasawa, Yukio Maezawa, Kyohei Kanematsu, Takanobu Yamada, Norio Yukawa, Aya Saito, Takashi Ogata, Takashi Oshima

**Affiliations:** 1 Department of Gastrointestinal Surgery, Kanagawa Cancer Center, 2-3-2 Nakano Asahi-ku, Yokohama 241-8515, Kanagawa, Japan; hashimoto.ita.ic@yokohama-cu.ac.jp (I.H.);; 2 Department of Surgery, Yokohama City University, 3-6 Fukuura Kanazawa-ku, Yokohama 236-0004, Kanagawa, Japan

**Keywords:** gastric cancer, sarcopenic obesity, prognosis

## Abstract

**Background/Objectives:** Preoperative body composition has been implicated as a factor affecting clinical outcomes in several types of cancer. However, there is limited evidence regarding whether preoperative body composition can predict the prognosis following gastrectomy for gastric cancer (GC). We aimed to investigate the role of preoperative body composition as a prognostic factor for overall survival (OS) and relapse-free survival (RFS) after gastrectomy for GC. **Methods:** This prospective study included 540 patients who underwent gastrectomy for GC at the Kanagawa Cancer Center, Japan, between December 2013 and November 2017. Preoperative body composition was assessed using the skeletal muscle index and visceral adipose tissue area derived from computed tomography scans. Patients were classified into four groups: non-sarcopenic non-obesity (NN), sarcopenic non-obesity (SN), non-sarcopenic obesity (NO), and sarcopenic obesity (SO). **Results:** A total of 448 patients (NN, 184; SN, 52; NO, 186; SO, 26) were included in the final analysis. In terms of OS, the SO group showed significantly worse survival than the NN group (72.1% vs. 87.6%, *p* = 0.01). Similarly, regarding RFS, the SO group had significantly worse outcomes than the NN group (68.4% vs. 86.2%, *p* = 0.007). Multivariate analysis identified SO as an independent risk factor for both OS (hazard ratio [HR], 3.18; 95% confidence interval [CI], 1.33–7.64; *p* = 0.01) and RFS (HR, 3.08; 95% CI, 1.36–6.95; *p* = 0.01). **Conclusions:** Preoperative SO was associated with poorer outcomes in patients undergoing gastrectomy for GC.

## Simple Summary

Sarcopenic obesity (SO) refers to the coexistence of low muscle mass and excess visceral fat. However, its prognostic impact in patients undergoing gastrectomy for gastric cancer remains unclear. In this study, preoperative body composition was assessed using computed tomography, and patients were classified into four groups based on skeletal muscle mass and visceral fat. Patients with SO showed significantly worse overall survival and relapse-free survival than those in other groups, even after adjustment for clinicopathological factors.

## 1. Introduction

Gastric cancer (GC) remains one of the leading causes of cancer-related deaths worldwide despite recent advances in surgical techniques, perioperative care, and systemic therapies [1,2]. In recent years, body composition parameters—particularly sarcopenia, which is defined as the loss of skeletal muscle (SM) mass and function—have gained attention as important prognostic indicators in various malignancies [3,4], including GC [5,6]. Similarly, visceral obesity, which reflects an increase in visceral adipose tissue (VAT), has been associated with poor prognosis owing to its pro-inflammatory and immunosuppressive effects [7,8,9] in GC.

Sarcopenic obesity (SO), characterized by the coexistence of sarcopenia and visceral obesity, represents a particularly harmful phenotype that combines the negative effects of both muscle wasting and excess adiposity [10]. Although the individual prognostic impact of SO in various cancers has been investigated in previous studies [11,12,13], its significance in patients undergoing gastrectomy remains unclear.

Therefore, we aimed to evaluate preoperative body composition—specifically SM mass and VAT—using computed tomography (CT) and to assess the effect of SO on long-term outcomes in patients undergoing curative gastrectomy for GC.

## 2. Materials and Methods

### 2.1. Patients

**Figure 1:**
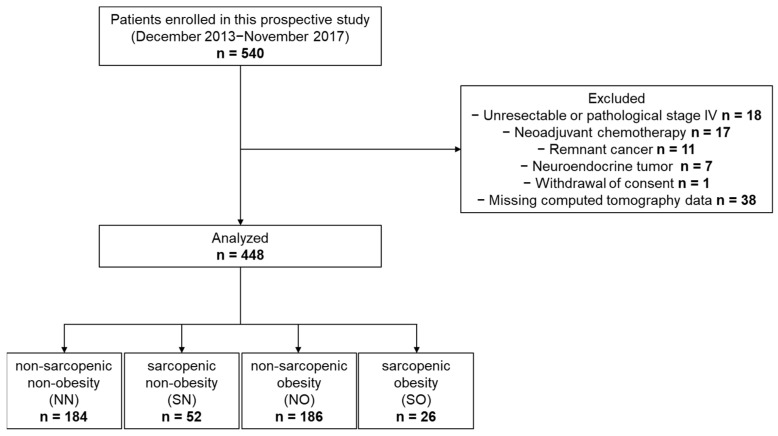
Flowchart of the patient selection process.

Between December 2013 and November 2017, a total of 540 patients with GC were enrolled in this prospective study. This study was based on a prospectively collected cohort, and the present analysis was conducted retrospectively. Owing to the limited availability of prior data on the prognostic significance of SO in GC, a formal sample size calculation was not performed. Instead, we determined the target sample size based on clinical and methodological considerations. Specifically, we aimed to include at least 150 patients undergoing total gastrectomy and at least 150 patients undergoing distal gastrectomy to ensure a balanced representation of surgical subtypes and facilitate exploratory subgroup analyses. The registration criteria were as follows: (i) GC proven using pathological diagnosis, (ii) gastrectomy achieved R0 resection as the initial treatment for GC, (iii) age > 20 years, and (iv) Eastern Cooperative Oncology Group performance status of 0–2. All included patients underwent preoperative CT. Ninety-two patients with missing CT data, neoadjuvant chemotherapy cases, remnant cancer, neuroendocrine tumor, or withdrawal of consent were excluded from this study. Therefore, 448 patients were analyzed in this study (Figure 1). All study protocols (25Research-20) were approved by the Ethics Committee of Kanagawa Cancer Center, and all procedures were conducted in accordance with the Declaration of Helsinki of 1996.

### 2.2. Image Analysis

**Figure 2:**
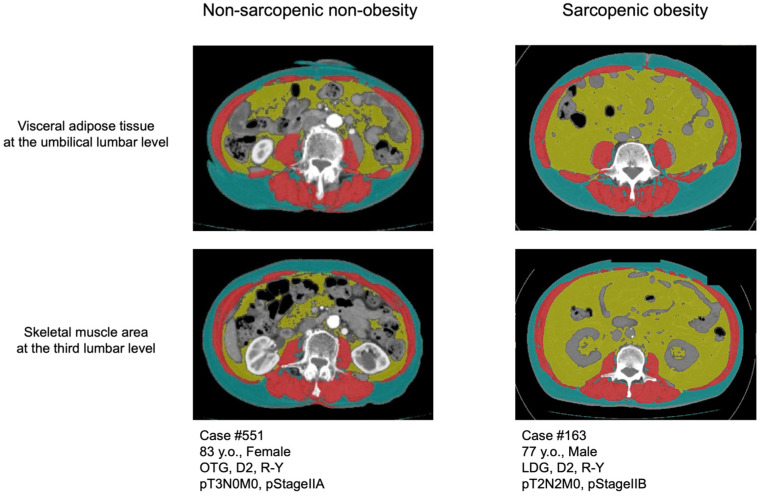
Representative computed tomography image of the skeletal muscle area (red) at the third lumbar level, visceral fat area (yellow), and subcutaneous fat area (green) at the umbilical lumbar level.

Preoperative CT was obtained within 60 days before surgery. We analyzed the SM at the third lumbar vertebra level and VAT at the umbilical level in the preoperative CT images (Aquillion 64; Toshiba Medical Systems, Otawara, Japan) using the graphics program SliceOmatic 5.0 Revision 9 (Tomovision, Magog, QC, Canada) and ABACS (Voronoi Health Analytics Inc., Vancouver, BC, Canada), which is an auto-segmentation module for muscle and adipose tissue [14,15]. All segmented images were reviewed by trained investigators, and manual corrections were performed when necessary to ensure accuracy. Reproducibility was confirmed through repeated assessments in a subset of cases. A threshold range of −29 to 150 HU was used to define SM, and a range of −150 to −50 HU was used to define VAT (Figure 2). Skeletal muscle index (SMI) was calculated by normalizing the cross-sectional area of the SM in centimeters squared by the height of the patient in meters squared. The cutoff values for SMI were defined as 40.31 cm^2^/m^2^ for males and 30.88 cm^2^/m^2^ for females [12,16]. Obesity was defined as a VAT area of ≥100 cm^2^ in both males and females [17]. Patients were categorized according to the presence or absence of sarcopenia and obesity into one of four body composition groups: non-sarcopenic non-obesity (NN), sarcopenic non-obesity (SN), non-sarcopenic obesity (NO), and SO. This study defines SO on the basis of CT-derived body composition parameters, rather than evaluating clinical SO.

### 2.3. Statistical Analysis

Categorical variables were evaluated using the chi-squared (χ^2^) test or Fisher’s exact test, as appropriate. Overall survival (OS) was defined as the time from surgery until death from any cause. Relapse-free survival (RFS) was defined as the time from surgery until the first documented recurrence or death from any cause. Cancer-specific survival (CSS) was defined as the time from surgery until death due to GC, with deaths from other causes being censored on the date of death. OS, RFS, and CSS were estimated using the Kaplan–Meier method, and differences between groups were compared using the log-rank test. Multivariable Cox proportional hazards models were fitted, including all prespecified covariates, in order to evaluate the factors associated with OS, RFS, and CSS. All prespecified covariates were included in the multivariable Cox models: age, sex, body mass index category, hypertension, diabetes mellitus, surgical procedure, tumor size, histological type, lymphatic invasion, venous invasion, pathological stage, postoperative complications and body composition group. The proportional hazards assumption was assessed using Schoenfeld residuals and no significant violations affecting the primary exposure were identified. A borderline deviation from the proportional hazards assumption was observed in the global test for RFS; however, no clear violation was detected for sarcopenic obesity, which was the primary exposure of interest. All statistical analyses were conducted using EZR (version 1.7; Saitama Medical Centre, Jichi Medical University, Saitama, Japan), a graphical user interface for R (version 4.5.2; The R Foundation for Statistical Computing, Vienna, Austria).

## 3. Results

### 3.1. Difference in Clinicopathological Factors Between the Body Composition Groups

During a median follow-up of 62.2 months, a total of 67 deaths, 42 recurrences, and 32 cancer-specific deaths were observed in the entire cohort. The median follow-up times were 62.4, 61.8, 61.8, and 63.0 months in the NN, SN, NO, and SO groups, respectively. During follow-up, 25, 7, 27, and 8 deaths occurred, with 20, 2, 17, and 3 recurrences and 16, 3, 11, and 2 GC–specific deaths in the NN, SN, NO, and SO groups, respectively. The association between preoperative body composition and clinicopathological factors in patients with GC is summarized in Table 1. A total of 448 patients were categorized as follows: NN (*n* = 184), SN (*n* = 52), NO (*n* = 186), and SO (*n* = 26). Significant differences in patient sex (*p* < 0.001), body mass index (*p* < 0.001), presence of hypertension (*p* = 0.02), diabetes mellitus (*p* = 0.03), and death due to other diseases (*p* = 0.01) were observed between the four body composition categories. A high proportion of patients in the SO group were male and had a normal body mass index, hypertension, diabetes mellitus, and death due to other diseases compared with patients in other groups.

### 3.2. Body Composition and Survival Outcomes

**Figure 3:**
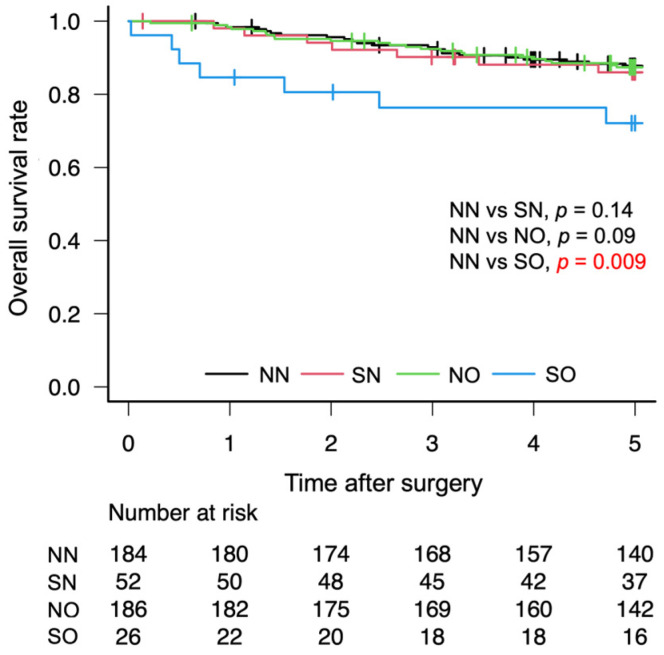
Five-year overall Kaplan–Meier survival curve according to body composition. Red letters indicate statistically significant *p*-values.

**Figure 4:**
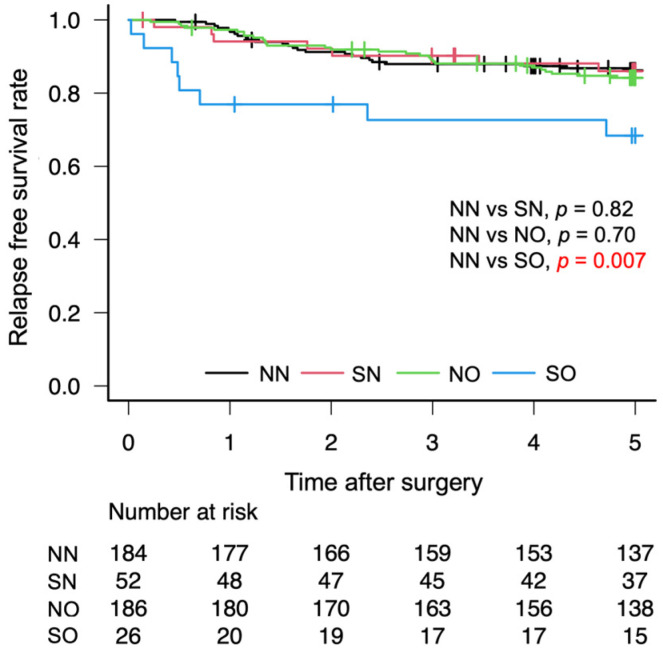
Five-year relapse-free Kaplan–Meier survival curve according to body composition. Red letters indicate statistically significant p-values.

**Figure 5:**
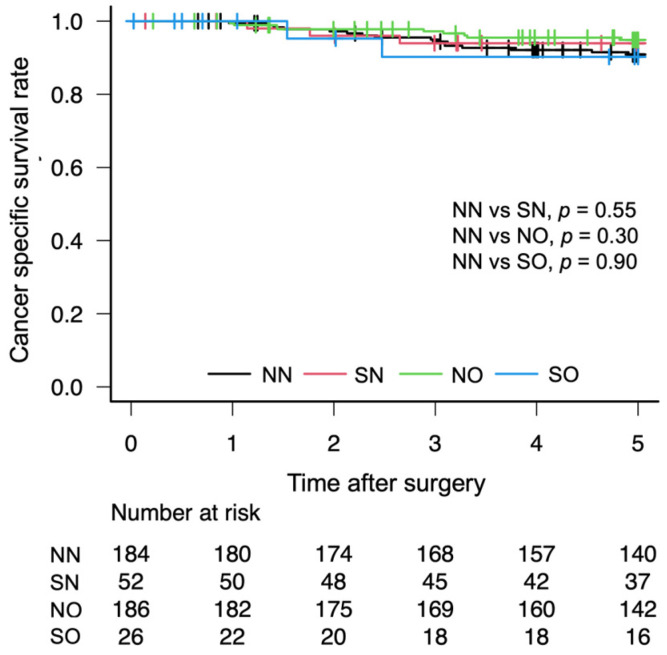
Five-year cancer-specific Kaplan–Meier survival curve according to body composition.

The OS rates after gastrectomy were lower in the SO group than in the NN group (72.1% vs. 87.6%, *p* = 0.01; Figure 3). The RFS rates after gastrectomy were lower in the SO group than in the NN group (68.4% vs. 86.2%, *p* = 0.01; Figure 4). There was no significant difference in the CSS rates between the SO and NN groups after gastrectomy (90.2% vs. 90.9%, *p* = 0.90; Figure 5).

### 3.3. Univariate and Multivariate Analyses for OS and RFS

The results of the univariate and multivariate analyses of OS, RFS, and CSS in patients with GC who underwent gastrectomy, stratified by body composition, are presented in Table 2, Table 3 and Table 4. Multivariate analysis identified SO as an independent prognostic factor for both OS (hazard ratio [HR], 3.18; 95% confidence interval [CI], 1.33–7.64; *p* = 0.01) and RFS (HR, 3.08; 95% CI, 1.36–6.95; *p* = 0.01).

## 4. Discussion

In this study, we examined the clinical significance of SO as a prognostic factor in patients with GC who underwent curative resection. Patients in the SO group had significantly worse OS and RFS than those in the NN group. Furthermore, we demonstrated that preoperative SO was an independent prognostic factor for poor prognosis in patients with GC who underwent curative resection.

Previous studies have shown that SO contributes to poor short-and long-term outcomes in various cancers [11,12,13]. Risk factors for postoperative complications and prognostic factors associated with long-term outcomes in GC have been investigated in numerous studies. Several retrospective studies have shown that SO is an independent risk factor for postoperative complications after gastrectomy  [18,19]. Furthermore, several studies have indicated that SO is an independent prognostic factor for long-term outcomes in patients with GC after gastrectomy [20]. Conversely, several studies have suggested that SO is not associated with a poor prognosis [19,21]. Although SO was a prognostic factor in our cohort, its prevalence was low, which is consistent with previous studies. Hence, large-scale prospective cohort studies are needed to understand the effects of SO on prognosis.

SO, characterized by low SM mass and excess visceral fat, is a high-risk condition in patients with GC. SO, leads to chronic inflammation, immune dysfunction, and metabolic changes, thereby reducing the antitumor effect and infection control [22,23,24]. High visceral fat also complicates surgery, increases operative stress, and delays wound healing, resulting in increased rates of infectious and cardiopulmonary complications [13,23,24,25,26]. Furthermore, patients with SO often have comorbidities such as hypertension and diabetes, which increase the perioperative risk and likelihood of non-cancer-related death [27,28]. Finally, previous reports have suggested that altered drug pharmacokinetics in patients with SO may increase chemotherapy toxicity [29,30,31]. Taken together, these factors may partly explain the association between SO and poor long-term outcomes in patients with GC.

In clinical practice, SO should be considered a modifiable risk factor for GC. CT-based body composition assessment can identify high-risk patients before surgery [30]. Once identified, both body composition and comorbidities must be targeted for management. Recently, growing evidence has supported prehabilitation—a structured exercise program with nutritional support—to mitigate SO and perioperative risk [32,33,34]. Furthermore, because patients with SO frequently have several comorbidities, integrating comorbidity control into perioperative pathways is essential, as it may improve treatment tolerance, reduce complications, and support overall prognosis [27,35]. In our cohort, non-cancer deaths were more common than cancer-related deaths; therefore, structured comorbidity management is warranted.

This study has several limitations. First, because no prior studies were available to estimate the effect size of SO in patients with GC, a formal sample size calculation was not performed, and the study was designed as an exploratory prospective cohort study. As a result, the statistical power to detect differences in outcomes involving the SO subgroup may have been limited, and the findings should be interpreted with caution. Second, although the data were prospectively collected, the analysis was performed at a single institution, which may have limited the external generalizability. Third, functional assessments such as grip strength or gait speed were not available, precluding a comprehensive evaluation of muscle quality. Therefore, SO in this study reflected a CT-defined condition rather than clinically diagnosed SO. Fourth, the cutoff values used to define sarcopenia and visceral obesity were based on Japanese population data and may not be applicable to other ethnicities. Given the relatively small number of patients with SO, additional sensitivity analyses using alternative cut-off values were not performed, as such analyses may have been underpowered and statistically unstable. Future studies with larger sample sizes are warranted to further validate and optimize clinically relevant cut-off values for body composition parameters in patients with GC.

## 5. Conclusions

In conclusion, preoperative SO was independently associated with poor OS and RFS in patients who underwent gastrectomy for GC. Incorporating CT-based body composition analysis into routine preoperative assessments may enhance risk stratification and inform individualized perioperative management strategies aimed at improving patient outcomes.

## Figures and Tables

**Table 1:** Clinicopathological data of patients in this study.

Variables	All Patients	NN	SN	NO	SO	*p*-Value
	(*n* = 448)	(*n* = 184)	(*n* = 52)	(*n* = 186)	(*n* = 26)	
Age	<65	61 (33.2)	15 (28.8)	58 (31.2)	5 (19.2)	0.533
	≥65	123 (66.8)	37 (71.2)	128 (68.8)	21 (80.8)	
BMI	<18.5	29 (15.8)	16 (30.8)	0 (0.0)	1 (3.8)	<0.001
	≥18.5, <25.0	145 (78.8)	36 (69.2)	100 (53.8)	24 (92.3)	
	≥25	10 (5.4)	0 (0.0)	86 (46.2)	1 (3.8)	
Hypertension	-	128 (69.6)	36 (69.2)	102 (54.8)	14 (53.8)	0.015
	+	56 (30.4)	16 (30.8)	84 (45.2)	12 (46.2)	
Diabetes mellitus	-	171 (92.9)	47 (90.4)	155 (83.3)	22 (84.6)	0.033
	+	13 (7.1)	5 (9.6)	31 (16.7)	4 (15.4)	
COPD	-	156 (84.8)	40 (76.9)	148 (79.6)	18 (69.2)	0.186
	+	28 (15.2)	12 (23.1)	38 (20.4)	8 (30.8)	
Operation	not TG	142 (77.2)	38 (73.1)	140 (75.3)	20 (76.9)	0.931
	TG	42 (22.8)	14 (26.9)	46 (24.7)	6 (23.1)	
Tumor size	≤30	97 (52.7)	26 (50.0)	96 (51.6)	15 (57.7)	0.927
	>30	87 (47.3)	26 (50.0)	90 (48.4)	11 (42.3)	
Histological type	Well/Moderately	77 (41.8)	29 (55.8)	101 (54.3)	14 (53.8)	0.071
	Poorly	107 (58.2)	23 (44.2)	85 (45.7)	12 (46.2)	
Lymphatic invasion	-	123 (66.8)	36 (69.2)	135 (72.6)	19 (73.1)	0.663
	+	61 (33.2)	16 (30.8)	51 (27.4)	7 (26.9)	
Venous invasion	-	111 (60.3)	32 (61.5)	108 (58.1)	15 (57.7)	0.954
	+	73 (39.7)	20 (38.5)	78 (41.9)	11 (42.3)	
pStage	I	119 (64.7)	40 (76.9)	135 (72.6)	17 (65.4)	0.221
	II–III	65 (35.3)	12 (23.1)	51 (27.4)	9 (34.6)	
Surgical complications	-	161 (87.5)	45 (88.2)	148 (79.6)	22 (88.0)	0.143
	+	23 (12.5)	6 (11.8)	38 (20.4)	3 (12.0)	
Death due to other diseases	-	175 (95.1)	48 (92.3)	170 (91.4)	20 (76.9)	0.013
	+	9 (4.9)	4 (7.7)	16 (8.6)	6 (23.1)	

BMI, body mass index; COPD, chronic obstructive pulmonary disease; NN, non-sarcopenic non-obesity; NO, non-sarcopenic obesity; pStage, pathological stage; SN, sarcopenic non-obesity; SO, sarcopenic obesity; TG, total gastrectomy.

**Table 2:** Univariate and multivariate analyses of clinicopathological factors and body composition for overall survival.

Factors		Univariate		*p*-Value	Multivariate		*p*-Value
		HR	95% CI		HR	95% CI	
Age	<65	1			1		
	≥65	1.98	1.08–3.62	0.03	1.88	0.99–3.55	0.05
BMI	<18.5	1			1		
	≥18.5, <25.0	0.73	0.34–1.55	0.41	0.55	0.24–1.25	0.16
	≥25	1.01	0.44–2.32	0.98	0.74	0.26–2.12	0.57
Hypertension	-	1			1		
	+	1.27	0.78–2.07	0.34	0.96	0.57–1.61	0.87
Diabetes mellitus	-	1			1		
	+	1.57	0.82–3.00	0.18	1.79	0.91–3.56	0.09
Operation	not TG	1			1		
	TG	2.02	1.23–3.32	0.01	1.69	0.99–2.88	0.05
Tumor size	≤30	1			1		
	>30	1.88	1.14–3.09	0.01	0.85	0.47–1.52	0.57
Histological type	Well/Moderately	1			1		
	Poorly	1.21	0.74–1.96	0.45	1.13	0.68–1.87	0.65
Lymphatic invasion	-	1			1		
	+	3.33	2.04–5.42	<0.001	1.81	1.03–3.17	0.04
Venous invasion	-	1			1		
	+	4.01	2.35–6.83	<0.001	1.93	1.02–3.64	0.04
Stage	I	1			1		
	II–III	4.76	2.87–7.91	<0.001	2.87	1.46–5.63	0.002
Surgical complications	-	1			1		
	+	0.79	0.39–1.60	0.52	0.68	0.33–1.40	0.30
Body composition	NN	1			1		
	SN	1.07	0.46–2.48	0.88	1.29	0.54–3.09	0.57
	NO	1.09	0.63–1.90	0.75	1.22	0.60–2.48	0.58
	SO	2.75	1.23–6.12	0.01	3.18	1.33–7.64	0.01

CI, confidence interval; HR, hazard ratio.

**Table 3:** Univariate and multivariate analyses of clinicopathological factors and body composition for relapse-free survival.

Factors		Univariate		*p*-Value	Multivariate		*p*-Value
		HR	95% CI		HR	95% CI	
Age	<65	1			1		
	≥65	1.80	1.03–3.12	0.04	1.66	0.93–2.96	0.09
BMI	<18.5	1			1		
	≥18.5, <25.0	0.71	0.35–1.45	0.35	0.55	0.25–1.20	0.13
	≥25	1.08	0.50–2.35	0.84	0.83	0.31–2.23	0.72
Hypertension	-	1			1		
	+	1.32	0.84–2.08	0.23	1.03	0.64–1.66	0.91
Diabetes mellitus	-	1			1		
	+	1.47	0.79–2.72	0.22	1.64	0.86–3.15	0.14
Operation	not TG	1			1		
	TG	1.80	1.13–2.88	0.01	1.50	0.91–2.47	0.11
Tumor size	≤30	1			1		
	>30	1.99	1.25–3.17	0.004	1.01	0.59–1.74	0.97
Histological type	Well/Moderately	1			1		
	Poorly	1.24	0.79–1.96	0.34	1.15	0.72–1.86	0.56
Lymphatic invasion	-	1			1		
	+	3.40	2.16–5.35	<0.001	2.10	1.24–3.55	0.01
Venous invasion	-	1			1		
	+	2.96	1.85–4.74	<0.001	1.34	0.76–2.38	0.31
Stage	I	1			1		
	II–III	4.29	2.70–6.82	<0.001	2.55	1.37–4.73	0.003
Surgical complications	-	1			1		
	+	0.96	0.52–1.79	0.90	0.89	0.47–1.67	0.71
Body composition	NN	1			1		
	SN	0.90	0.39–2.06	0.80	1.04	0.44–2.46	0.92
	NO	1.11	0.67–1.84	0.70	1.10	0.57–2.11	0.78
	SO	2.74	1.29–5.81	0.01	3.08	1.36–6.95	0.01

CI, confidence interval; HR, hazard ratio.

**Table 4:** Univariate and multivariate analyses of clinicopathological factors and body composition for cancer-specific survival.

Factors		Univariate		*p*-Value	Multivariate		*p*-Value
		HR	95% CI		HR	95% CI	
Age	<65	1			1		
	≥65	1.06	0.50–2.24	0.88	1.15	0.51–2.57	0.74
BMI	<18.5	1			1		
	≥18.5, <25.0	0.43	0.17–1.08	0.07	0.42	0.15–1.15	0.09
	≥25	0.58	0.20–1.68	0.32	0.63	0.15–2.60	0.52
Hypertension	-	1			1		
	+	1.32	0.84–2.08	0.23	1.00	0.47–2.14	0.99
Diabetes mellitus	-	1			1		
	+	1.47	0.79–2.72	0.22	1.51	0.48–4.68	0.48
Operation	not TG	1			1		
	TG	1.54	0.73–3.25	0.26	1.16	0.51–2.60	0.72
Tumor size	≤30	1			1		
	>30	4.09	1.77–9.45	<0.001	1.10	0.44–2.72	0.84
Histological type	Well/Moderately	1			1		
	Poorly	2.59	1.20–5.59	0.02	1.73	0.77–3.85	0.18
Lymphatic invasion	-	1			1		
	+	13.67	5.26–35.49	<0.001	4.02	1.45–11.12	0.007
Venous invasion	-	1			1		
	+	8.82	3.39–22.90	<0.001	1.75	0.61–5.01	0.30
Stage	I	1			1		
	II–III	38.56	9.21–161.40	<0.001	13.69	2.74–68.42	0.001
Surgical complications	-	1			1		
	+	0.71	0.25–2.03	0.52	0.62	0.21–1.88	0.40
Body composition	NN	1			1		
	SN	0.68	0.20–2.35	0.55	1.32	0.36–4.80	0.68
	NO	0.67	0.31–1.45	0.31	0.97	0.33–2.84	0.96
	SO	1.06	0.24–4.63	0.93	1.71	0.36–8.05	0.50

CI, confidence interval; HR, hazard ratio.

## Data Availability

The data presented in this study are available on request from the corresponding author due to ethical reasons.
